# 687. Use of Dalbavancin in Gram-positive Infective Endocarditis: Review of Current Literature

**DOI:** 10.1093/ofid/ofab466.884

**Published:** 2021-12-04

**Authors:** Tasaduq Fazili, Ekta Bansal, Mariana Gomez de la Espriella, Dorothy C Garner

**Affiliations:** 1 Virginia Tech Carilion School of Medicine, Roanoke, VA; 2 Carilion Clinic, Virginia Tech, Blacksburg, VA; 3 Virginia Tech Carilion School of Medicine, Carilion Clinic, Roanoke, VA

## Abstract

**Background:**

Dalbavancin is a long acting, semisynthetic derivative of teicoplanin that is currently approved for treatment of acute bacterial skin and skin structure infections. Its efficacy and role of in the treatment of invasive infections, in particular infective endocarditis, is not well known.

**Methods:**

We reviewed the English-language literature for the use of Dalbavancin in the treatment of endocarditis due to Gram-positive organisms, using Pubmed.

**Results:**

15 publications were reviewed. All the publications were retrospective in nature, with relatively small numbers of patients, including a few case reports.

A total of 159 patients received Dalbavancin for endocarditis. The mean age was 47 years. The main reasons for using Dalbavancin were non-feasibility of a standard outpatient regimen (mainly due to drug use) or the need for a simpler regimen. 75 patients had infection of a native valve, 44 of a prosthetic valve and 19 of a cardiac device. The type of infection for the rest of the patients was not specified. The tricuspid valve was the most frequently reported. The etiologic organisms causing endocarditis were Staphylococcus species, followed by Streptococcus species and Enterococcus species, with Staphylococcus aureus being the most common. All, but one, patients received Dalbavancin as sequential therapy, after receiving other intravenous antibiotics initially. The duration of antibiotics received prior to initiation of Dalbavancin was variable, with the median being 3 weeks. The median duration of Dalbavancin use was 2.7 weeks. The dosage regimens varied, with the more common ones using a loading dose of either 1500 mg or 1000 mg, followed by one or more weekly doses of 500 mg. The overall clinical efficacy was around 89%. Adverse events were mild, including nausea, vomiting, rash, headache and reversible acute kidney injury. None of the patients had to discontinue the drug because of adverse events. Two publications evaluated the cost effectiveness of Dalbavancin and found it to save about &9000 per patient, the saving being mainly due to reduced length of hospital stay.

**Conclusion:**

Dalbavancin appears to be an efficacious, safe and cost-effective option for sequential treatment of endocarditis caused by Staph aureus and other Gram-positive organisms.

**Disclosures:**

**All Authors**: No reported disclosures

